# Implication of high variance in germplasm characteristics

**DOI:** 10.1038/s41598-023-27793-z

**Published:** 2023-01-10

**Authors:** Ju-Kyung Yu, Sungyul Chang, Gyung Deok Han, Seong-Hoon Kim, Jinhyun Ahn, Jieun Park, Yoonha Kim, Jaeyoung Kim, Yong Suk Chung

**Affiliations:** 1 Seeds Research, Syngenta Crop Protection LLC, Research Triangle Park, Durham, NC 27709 USA; 2 Crop Protection & Physiology Division National Institute of Crop Science, RDA, Wanju, 55365 Republic of Korea; 3 Department of Practical Course Education, Cheongju National University of Education, Cheongju, 28708 Republic of Korea; 4 National Agrobiodiversity Center, National Institute of Agricultural Sciences (NAS), RDA, Jeonju, Republic of Korea; 5 Department of Management Information Systems, Jeju National University, Jeju, 63243 Republic of Korea; 6 Department of Plant Resources and Environment, Jeju National University, Jeju, 63243 Republic of Korea; 7 Department of Applied Biosciences, Kyungpook National University, Daegu, 41566 Republic of Korea; 8 Gene Engineering Division, National Institute of Agricultural Science, Jeonju, 54874 Republic of Korea

**Keywords:** Plant breeding, Plant ecology

## Abstract

The beauty of conserving germplasm is the securement of genetic resources with numerous important traits, which could be utilized whenever they need to be incorporated into current cultivars. However, it would not be as useful as expected if the proper information was not given to breeders and researchers. In this study, we demonstrated that there is a large variation, both among and within germplasm, using a low-cost image-based phenotyping method; this could be valuable for improving gene banks’ screening systems and for crop breeding. Using the image analyses of 507 accessions of buckwheat, we identified a wide range of variations per trait between germplasm accessions and within an accession. Since this implies a similarity with other important agronomic traits, we suggest that the variance of the presented traits should be checked and provided for better germplasm enhancement.

## Introduction

As the severity of climate change increases, it causes major challenges to crop production and negatively threatens the sustainability of global agriculture and food security. Breeding environment resilient crops is extremely urgent. Therefore gene banks play a critical role in conservation, harnessing the benefits of crop diversity, and providing raw breeding material containing desirable traits in order to ensure global agricultural sustainability^1^.

Currently, there are about 1750 gene banks globally, maintaining millions of crop accessions and their wild relatives; some examples are Germplasm Resources Information Network (GRIN) and the European Cooperative Programme for Plant Genetic Resources (ECPGR)^2^.

Gene banks are required to provide information regarding the accessions’ agronomic, physiological, and genetic traits; however, collecting and managing this information is costly and labor-intensive. Therefore, there is an inherent risk that plant information can be poorly managed at gene banks, especially in the case of underutilized crops^1,3^. For example, if accessions are not well purified and, as a result, higher heterogeneity dominates. Additionally, some plant phenotypic traits, even at gene banks that practice standard protocol, are based on visual evaluation (e.g., seed shape and color), and the descriptor of many traits are categorical by average value, which does not provide enough variance information^4^. Due to this, we probed how much variation could be found in seed traits of buckwheat as a case study. The purpose of the current study is to demonstrate that there is a large variation in the morphological traits of buckwheat seeds and, by extension, that variance information should be provided or checked before using germplasm.

## Results and discussion

Image-based technology has become one of the most promising in terms of cost, throughput, turnaround time, easy operation, and accuracy^5^. Additionally, it is a non-destructive technology and, as a result, is adopted quickly in plant sciences and breeding fields.

**Figure 1:**
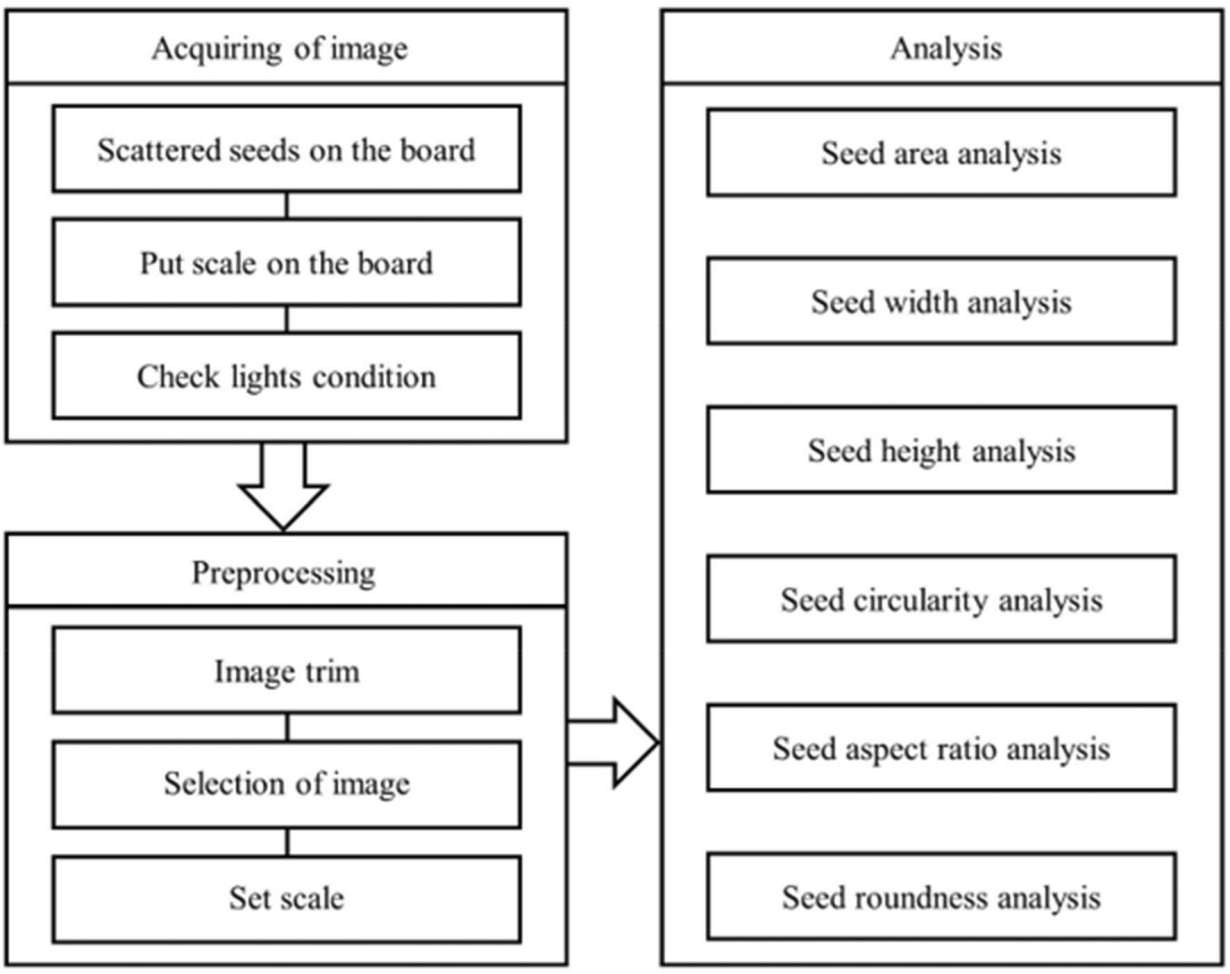
End to end workflow of high-throughput buckwheat seed phenotype analysis.

We show how the low-cost homemade image-based system that was implemented for this study can be effectively and efficiently adopted by investigators in resource-scarce research institutions and can be applied to collect and manage image data at a large-scale phenotyping operation at gene banks. The overall cost did not exceed $4,000 USD. System building took less than two business days. Figure 1 demonstrates the simple and straightforward steps of the phenotyping workflow: capturing the image, processing the image, and analyzing the data. The ImageJ software we used is open to the public. Learning these three steps took two business days, and the experiment was conducted in just five business days.

Buckwheat is a short-season crop that grows well in low-fertility soils, and its commodity is traded mainly within South Korea. Buckwheat noodles and flatbreads play a major role in Korean cuisine and get spotlighted as a gluten-free specialty crop. We image-screened the five most common traits of buckwheat seed shapes for 507 germplasm: seed area, width, height, circularity, and roundness. Detailed measurement descriptions are shown in Table 1.

**Table 1:** Parameter definition and Calculation method.

Parameter	Calculation method
Area	Area of selection in square pixels or in calibrated square units
Width	Width of the smallest rectangle enclosing the selection area
Height	Height of the smallest rectangle enclosing the selection area
Circularity	Circularity = 4ΠA/P^2^ with a value of 1.0 indicating a perfect circle. As the value approaches 0.0, it indicates an increasingly elongated shape. Values may not be valid for very small particles
Roundness	4 × [Area]/π × [Major axis]^2^

**Figure 2:**
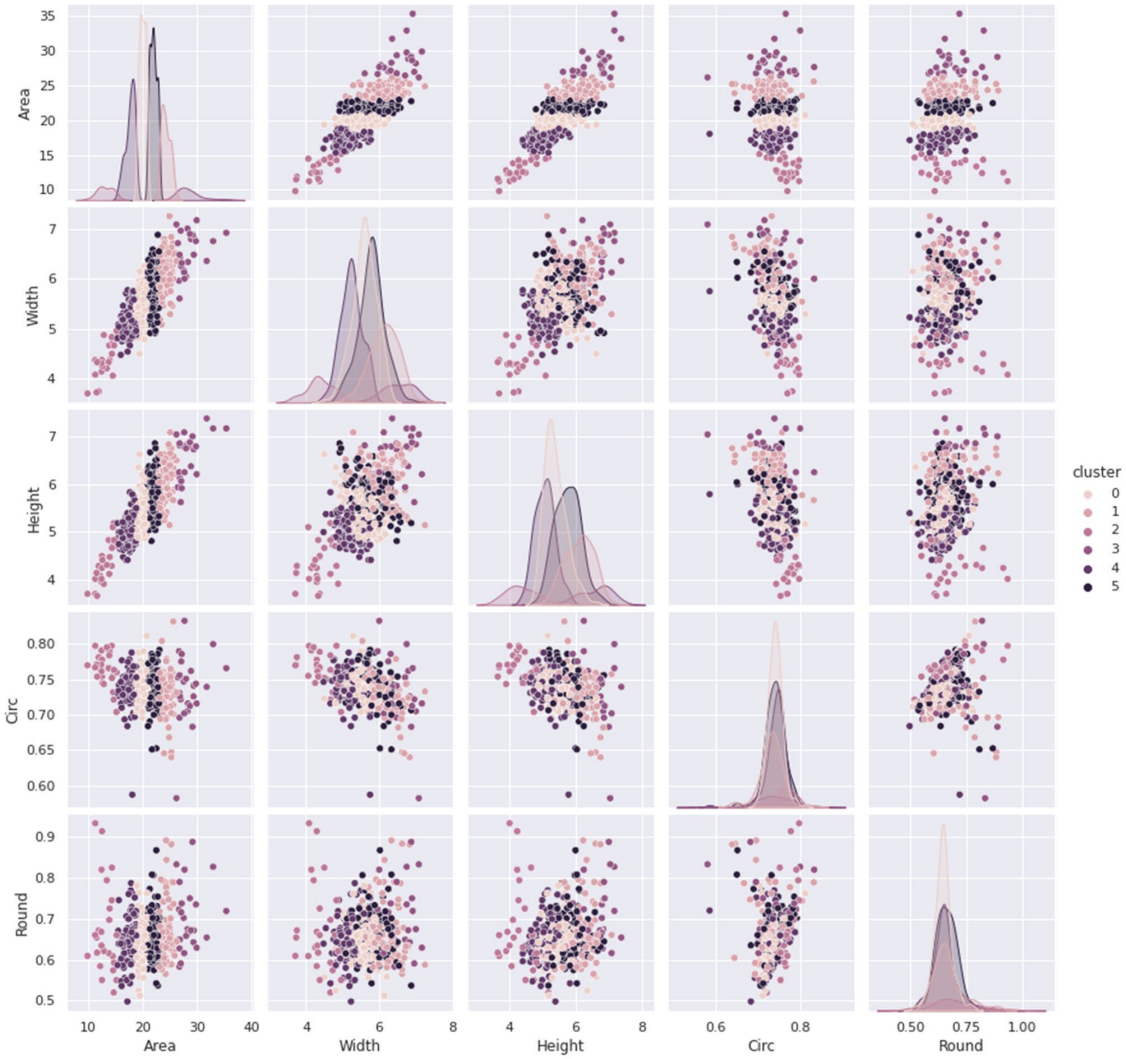
The pair plot of two feature combinations with 6 cluster labels.

The clustering analysis results of five traits based on k-means (k = 10) are shown in Fig. 2. Each trait shows significant variations among germplasm groups defined by k-means clustering analysis. It supports that image-based phenotyping could capture germplasm variation efficiently enough to discern the differences. It also demonstrates different levels of germplasm variations within each group. For example, the shapes of distribution (traits of seed area and width) are varied; some groups are in a smooth bell shape, but both traits also display multiple peaks indicating symmetrical or asymmetrical distribution shapes. For traits under investigation, we found an abundant variation within germplasm. For germplasm management, the standard protocol to collect seed shape information is based on visual evaluation; however, the descriptor is categorical of average value, which does not capture variations adequately. The variations revealed by image-based technology could enrich and strengthen the current gene bank database, which may create opportunities to utilize newly-discovered and beneficial alleles. This information on variation could be valuable for further crop breeding; however, under current systems, this information is absent^4^. Therefore, we urge gene banks to consider gathering and maintaining supplementary information by adopting image-based phenotyping technology to capture variations adequately.

**Figure 3:**
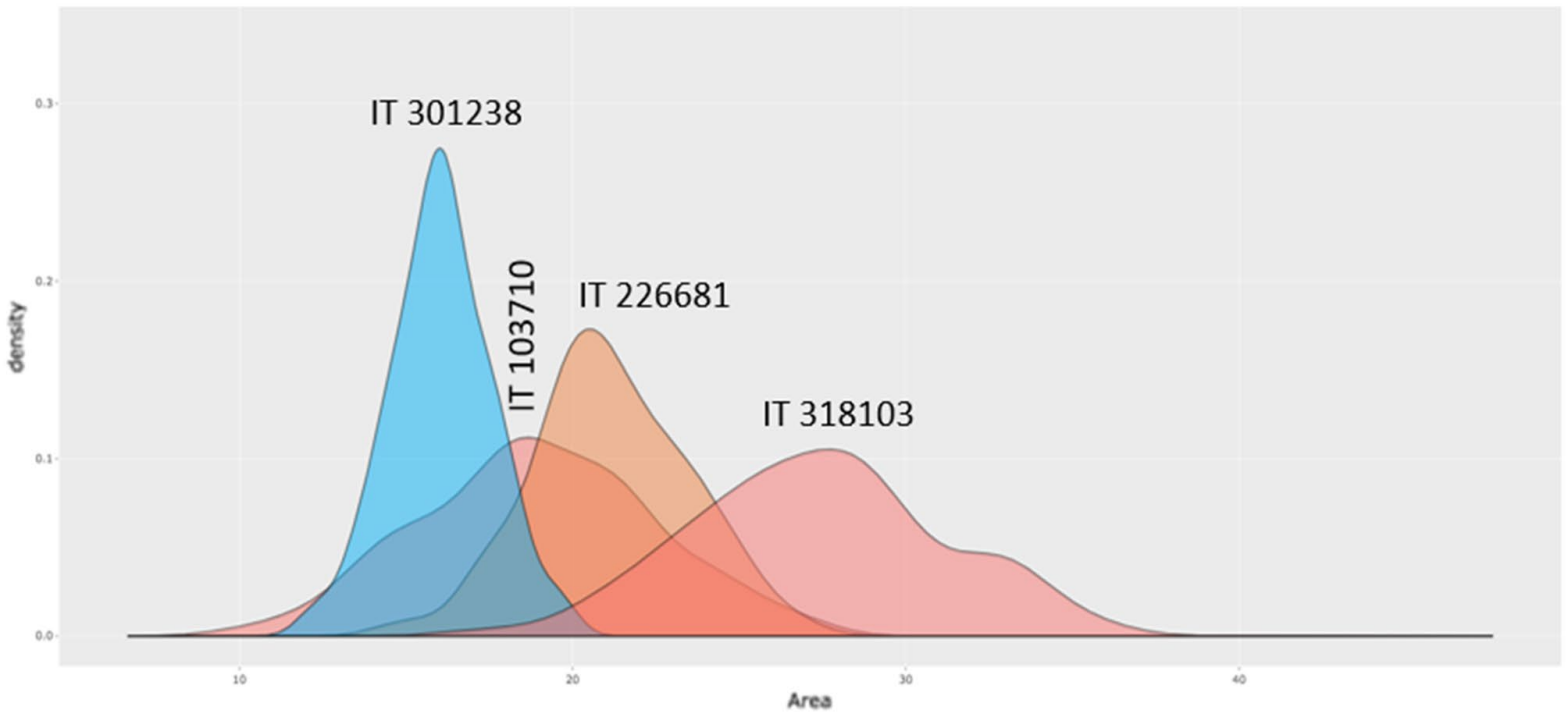
Feature (Area) density plot of four line (IT 301243, IT 103710, IT 226681 and IT 318103).

For germplasm in gene banks, we expect seeds of the same germplasm accession to be uniform in terms of morphology and genetics. However, the reality is often not what we expect, especially in commercially underutilized crops. There are a few explanations: (1) underutilized crops have received limited attention by the scientific community and industry, and (2) lack of infrastructure and resources to monitor incoming germplasm and maintain their uniformity at the site of the gene bank. If a breeder works in a well-funded institute or industry, they perform seed purification steps on incoming germplasm in the field or a greenhouse to ensure genetic uniformity before utilizing the germplasm for downstream activities. We evaluated 507 buckwheat germplasm accessions provided by the Rural Development Administration (RDA) in South Korea. Figure 3 shows the kernel density estimation plot of seed area trait for randomly selected of four accessions. The range of seed area value of accession IT301238 has the smallest variability while IT318103 has the largest variability. We speculate the seed uniformity of IT318103 is insufficient compared to the accessions IT378103 and IT226681. We observed several similar cases in germplasm screening studies, such as the soft rot study in wild potato germplasm^6^.

This information on variation within an accession may show that other morphologies would be simirnar story. Figure 3 shows the kernel density estimation plot of seed area trait for randomly selected four accessions. The range of seed area value of accession IT301238 has the smallest variability while IT318103 has the largest variability. We speculate the seed uniformity of IT318103 is insufficient compared to the accession IT378103 and IT226681. We observed several similar cases in germplasm screening studies, such as the soft rot study in wild potato germplasm^6^. Considering those variances among traits measured in the current study are independent each other, it could be assumed that other traits could have different level of variances within and among germplasm.

It also can be useful for germplasm management. Gene banks abiding by the routine practice when they acquire germplasm ensures that complete pedigree details and morphological data are included. However, it does not always guarantee the genetic homogeneity of the seeds per each accession. Nonetheless, simple seeds image data with variance information can be useful for gene bank staff when prioritizing which accessions may need to be purified in order to guarantee the homogeneity of the accession before making official documentation. Additionally, it can assist with identifying which accessions need extra attention when the seeds are reproduced for seed increase (bulk seeds increase vs. single seed descent increase).

Breeders have implemented different strategies to prioritize potential valuable accessions from gene banks which can be utilized for crop breeding by using phenotypes and associated genetic information. If the gene bank generates and maintains this variance information, it can be shared with breeders who request seeds of the accession. It would be applicable information for breeders to plan if they go through the seed purification process in their organization before using it for a breeding program or provide additional information to articulate a breeding strategy for harnessing the potential of genetic resources.

## Materials and methods

### Plant materials

A total of 507 buckwheat (*Fagopyrum esculentum*) germplasm accessions were provided by the Rural Development Administration (RDA) genebank, South Korea.

### Camera system setting

A complementary metal–oxide–semiconductor (CMOS) image sensor color camera (Nikon D7500, Nikon Imaging Japan Inc., Tokyo, Japan) with a resolution of 23.5 × 15.7 mm and lens (af-s dx Nikkon 16–80 mm f/2.8–4 e ed vr, Nikon Imaging Japan Inc., Tokyo, Japan) was used to acquire images. A studio box (M80 Studio, China) with a size of 800 × 800 × 800 mm was set up. A Light-Emitting Diode (LED) board (ArtLight, Unclepen co., Bucheon, Korea) with an area of 670 × 470 × 20 mm was set to reduce image error caused by shadows during data production. The shadows of buckwheat seeds were removed using a backlight. Also, two light boards sized 600 × 10 mm, with 5500 K ± 200 temperature and two LED lighting stands (N-T96 LED, Prodean co., Seoul, Korea) with 5600 K temperature were installed to remove the shadows of buckwheat seeds during the process of taking images in the studio box. Buckwheat seeds were manually spread on the area of blue polypropylene (PP) (color clear PP “L” Holder, Hyunpoong Inc. co. Pochen. Korea), which was 255 × 310 mm. The blue PP had a chroma-key effect, making it easy to separate buckwheat seeds from the background. Each image taken by this system contains 95 seeds per germplasm accession on average.

We acquired vertical red, green, and blue (RGB) images of buckwheat seeds taken 25 cm above the ground with the camera (Nikon, Japan). In order to calculate the data compared to the actual size, a 16 mm tag was used as a scale bar. In order to minimize the error of the color value depending on the light condition, a standard color was selected, and a color tag was added to the blue PP. Pictures of the camera setting and seed image, as an example, can be found in Supplementary Figs. 1 and 2.

### Image analysis processing

The buckwheat seed images were processed by a software developed in Rural Development Administration in Republic of Korea based on the program ImageJ (ImageJ, National Institutes of Health, USA, rsd.info.nih-gov/ij). The program has allowed us to edit, calibrate, measure, analyze, and process image data^7^. It could be extended as a tool such as macro, which was used with ImageJ in the experiment. While editing images, we converted the size to millimeters in the scale setting. The standard tag was selected and set to the size of 16 mm for the pixel.

In order to separate into RGB channels, ImageJ, which was used to split seeds from the background, was used. The separation of RGB channels made it easier to separate the seeds from the background because the color of the seeds was simplified. After the separation of RGB channels, binary images were created by using threshold values of pixel values to complete the separation of seeds and background. The noise particles were processed at a pixel value of 100 times smaller than the size of the seeds to avoid the measurement of noise particles other than buckwheat seeds in the image. The area of buckwheat seed was separated into each of the parts as an independent area without connecting the objects^8^. Figure 1 outlines the end-to-end pipeline of the image-based phenotyping.

### Data analysis

Five seed shape characteristics were imaged: seed area, width, height, circularity, and roundness (Table 1). The characteristics were extracted from the images of individual buckwheat seeds (Table 1). All data analysis were perform using Python 3.8.5^9^. The data consists of 48,047 samples with 507 IT lines. The average, maximum, and minimum numbers of samples per line are 94.7, 238 and 41, respectively. We removed four samples with zero values and one having a height of 99.163, which is too large as the average height was 5.67. Data distribution of each feature per line was tested using the Shapiro–Wilk normality test^10^. 507 lines failed due to the fact that at least one of the features in the line has the p-value less than 0.05. In order to further check the data distribution, each feature in selected buckwheat lines was visualized with kernel density estimate (KDE) plots in Seaborn package (See Fig. 1). X-axis indicates ranges of data in a single feature. Y-axis indicates the probability of density that can be viewed as smoothing histograms. Even if the normality test failed, we can see that the data approximately follows the normal distribution, and no outlier exists.

The median value of each feature per line was calculated for clustering. The k-means clustering was selected because it showed robust clustering results in various data sets^11^. For a given k, k-means clustering partitions samples into k clusters in which each sample belongs to the cluster with the nearest distance. The k-means clustering tries to minimize the distortion, which is the sum of the squared distances between each sample and its centroid. Supplementary Fig. 3 shows the calculated distortion value according to the number of clusters.

The distortion value decreases dramatically until k is smaller than 6. For the larger k, the distortion value does not decrease dramatically. The optimal choice of k would be 6. Supplementary Fig. 3 visualizes the result of K-means clustering when k is 6 for pairs of two features located in X and Y axis. The plots on the diagonal show the density distribution of a corresponding feature for each cluster.

The kernel density estimate (KDE) plot is widely utilized to visualize various data types and easily visualize the peak of data in the intervals^12^. The density plot of each feature in the selected four accessions was visualized with the Plotly (Fig. 3). The x-axis indicated ranges of data in a single feature. The y-axis indicated the probability of density that corresponded to the x-axis, and it could be more significant than one^13^.In addition, the density plot of each feature per all accessions can be found in Supplementary Fig. 4.

We calculated the correlations between features using Spearman’s method^14^ (See Fig. 2). The method was selected due to the fact that the data did not fully follow the normality distribution. Based on the correlation coefficient and the p-value (Fig. 3), we can confirm that there are no correlations between features.

## Supplementary Information

Supplementary Figures.

## Data Availability

The datasets generated and/or analysed during the current study are not publicly available because mateirals were provided from Rural Development Administration (RDA) genebank but are available from the corresponding author on reasonable request.
