# Supplementary material for: Implication of high variance in germplasm characteristics
Source: Sci Rep. 2023 Jan 10;13:515. doi: 10.1038/s41598-023-27793-z (PMC9832015; doi:10.1038/s41598-023-27793-z)

**Supplement Figure 1.** Picture of camerasss setting


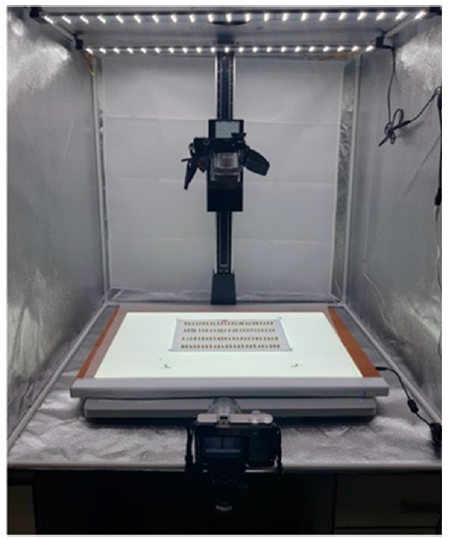


**Supplement Figure 2.** Data conversion from image to digital figures


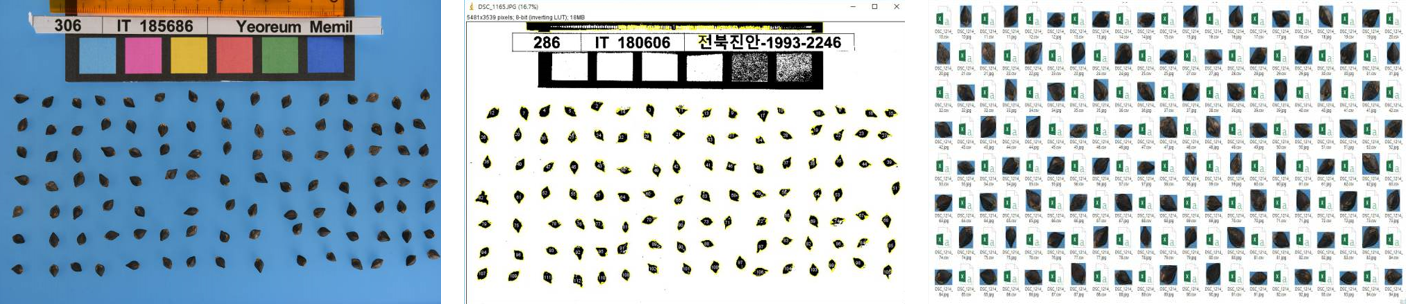


**Supplement Figure 3.** The distortion value varying the number of clusters
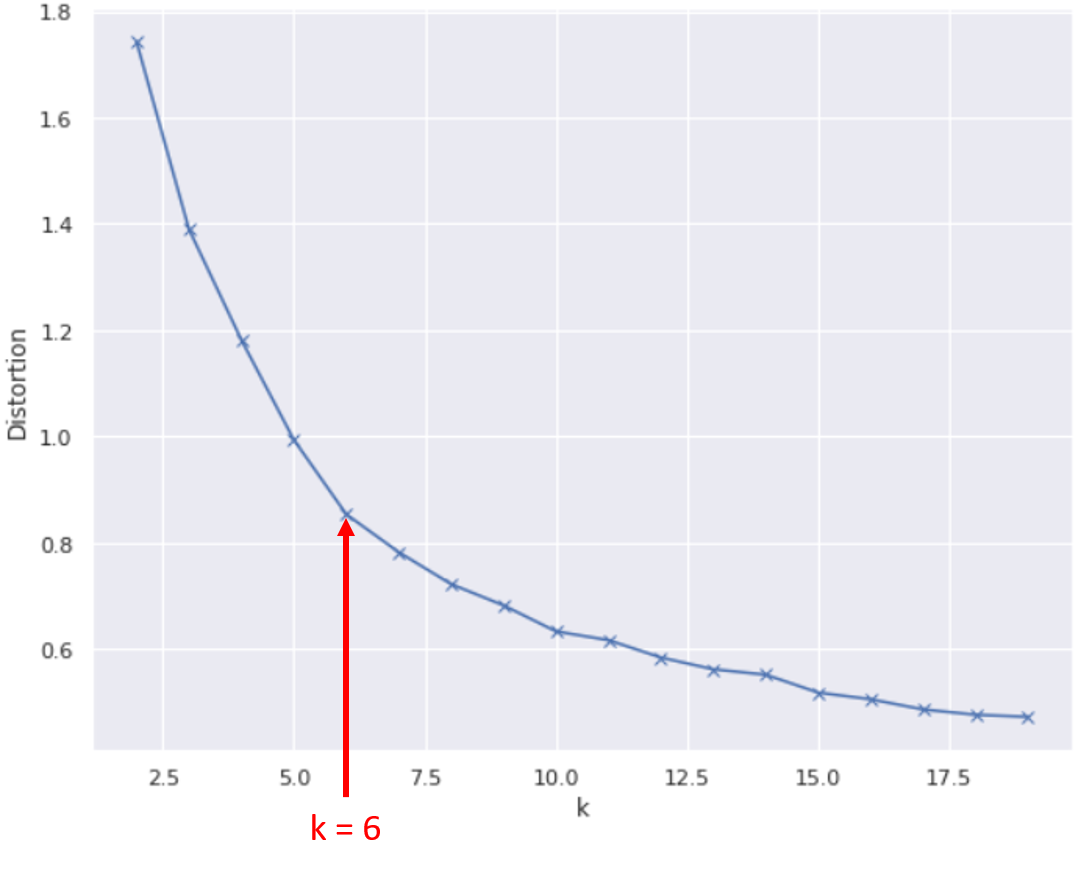


**Supplement Figure 4.** The kernel density estimate plots for each variables


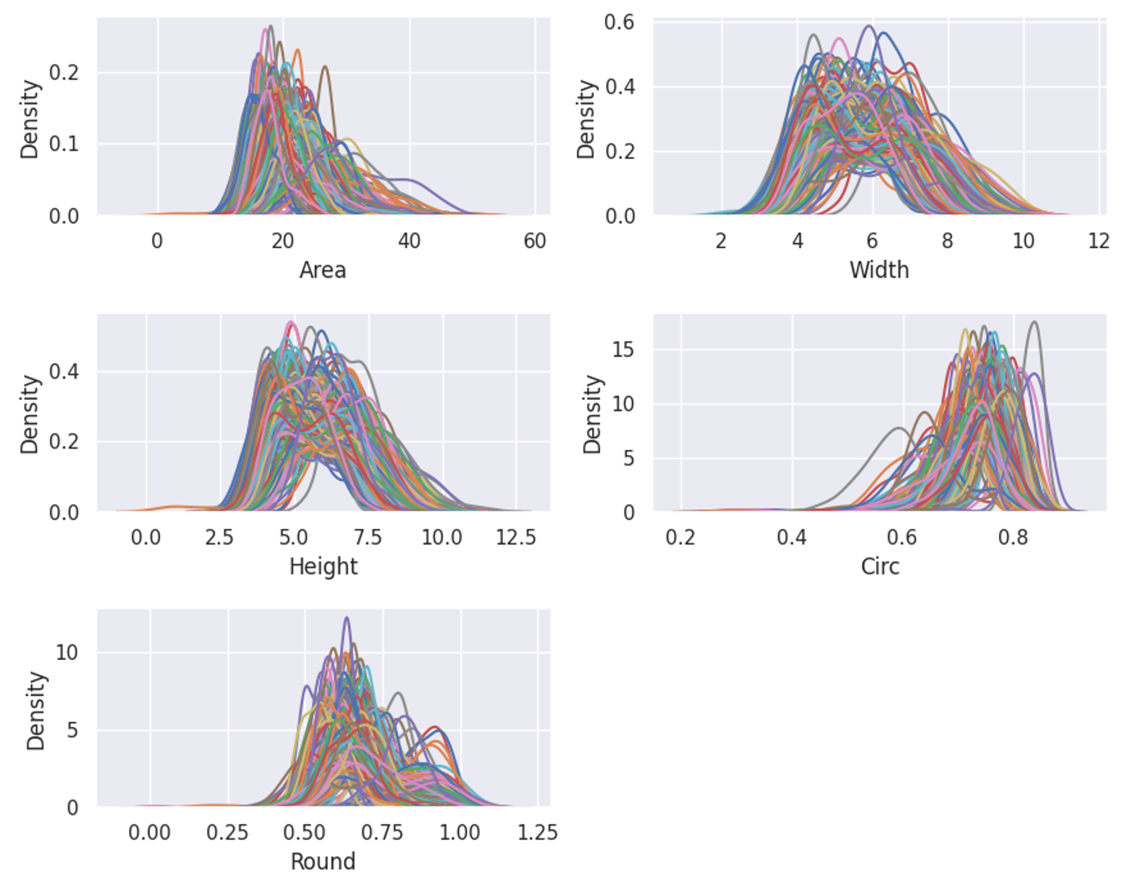

Supplement: Supplementary file 1 — Supplementary Figures. [file 41598_2023_27793_MOESM1_ESM.docx]
